# MRI characterization of focal liver lesions in non-cirrhotic patients: assessment of added value of gadoxetic acid-enhanced hepatobiliary phase imaging

**DOI:** 10.1186/s13244-020-00894-3

**Published:** 2020-09-22

**Authors:** Bardia Moosavi, Anuradha S. Shenoy-Bhangle, Leo L. Tsai, Robert Reuf, Koenraad J. Mortele

**Affiliations:** 1 Department of Radiology, Hull Hospital, Gatineau, Quebec J8Y1W7 Canada; 2 Division of Abdominal Imaging, Department of Radiology, Beth Israel Deaconess Medical Center, Harvard Medical School, 330 Brookline Ave, Boston, MA 02215 USA

**Keywords:** Liver tumor, Magnetic resonance imaging, Gadoxetic acid, Hepatospecific, Contrast media

## Abstract

**Background:**

To evaluate the added value of the hepatobiliary (HPB) phase in gadoxetic acid-enhanced magnetic resonance imaging (MRI) in characterizing newly discovered indeterminate focal liver lesions in non-cirrhotic patients.

**Results:**

One-hundred and twenty-five non-cirrhotic patients (median age, 46 years; range, 20–85 years; 100 females) underwent gadoxetic acid-enhanced MRI, including the 20-min delayed HPB phase, for characterization of newly discovered focal liver lesions. Images were independently evaluated by two blinded, board-certified abdominal radiologists (R1 and R2) who characterized liver lesions without and with assessment of the HPB phase images in two separate readout sessions. Confidence in diagnosis was scored on a scale from 0 to 3. Inter-observer agreement was assessed using Cohen κ statistics. Change in diagnosis and confidence in diagnosis were evaluated by Wilcoxon signed rank test. There was no significant change in diagnosis before and after evaluation of the HPB phase for both readers (*p* = 1.0 for R1; *p* = 0.34 for R2). Confidence in diagnosis decreased from average 2.8 ± 0.45 to 2.6 ± 0.59 for R1 and increased from 2.6 ± 0.83 to 2.8 ± 0.46 for R2. Change in confidence was only statistically significant for R1 (*p* = 0.003) but not significant for R2 (*p* = 0.49). Inter-reader agreement in diagnosis was good without (*k* = 0.66) and with (*k* = 0.75) inclusion of the HPB phase images.

**Conclusions:**

The added information obtained from the HPB phase of gadoxetic acid-enhanced MRI does not change the diagnosis or increase confidence in diagnosis when evaluating new indeterminate focal liver lesions in non-cirrhotic patients.

## Key points

- Gadoxetic acid not necessary for MR characterization of indeterminate lesions in non-cirrhotic liver.
- No new information from the HPB phase compared with conventional dynamic post-contrast sequences.
- No significant change in diagnosis based on MR contrast mixture

## Background

Characterization of focal liver lesions detected on ultrasound (US) or computed tomography (CT) is a common indication for magnetic resonance imaging (MRI) of the liver. Accurate characterization of focal liver lesions is necessary because management differs not only between benign and malignant lesions but also between different benign lesions [1]. For example, a commonly encountered clinical scenario is the differentiation between focal nodular hyperplasia (FNH) from hepatocellular adenoma (HCA). FNH and HCA occur in similar patient populations and have overlapping imaging features at MRI [2, 3], yet management of these conditions is considerably different [4]. The use of gadolinium-containing contrast agents with both extracellular blood pool and hepatocyte-specific properties has been advocated in addressing this clinical problem among others because it is thought to add new information in characterization [5]. Some of the drawbacks associated with the use of gadoxetic acid include increased scan time, relatively increased cost, and higher relaxivity which in turn implies higher molecular weight and increased protein bonding contributing to some of the side effects including transient dyspnea seen in up to 10% of patients, particularly those with chronic obstructive pulmonary disease, occasional headache, and dizziness [6, 7]. In a large trial, gadoxetic acid was shown to have high diagnostic accuracy in differentiating FNH from HCA [8]. However, more recent studies have suggested that the previously reported accuracy of gadoxetic acid-enhanced MR may be an overestimate [9, 10]. Furthermore, small-sized studies by Donati et al. and Purysko et al. have shown that the addition of the 20-min delayed hepatobiliary (HPB) phase in gadoxetic acid-enhanced MR imaging does not improve diagnostic accuracy for characterizing primary liver tumors in non-cirrhotic patients despite an overall increase in reader confidence in diagnosis [11, 12].

The purpose of our study was, therefore, to evaluate the added value of the HPB phase gadoxetic acid-enhanced MRI in characterizing newly discovered indeterminate focal liver lesions in a large cohort of non-cirrhotic patients by assessing its impact on diagnostic accuracy and the level of diagnostic confidence.

## Methods

### Subjects

This retrospective Health Insurance Portability and Accountability Act (HIPPA)-compliant study was approved by our institutional review board and informed patient consent was waived.

**Fig. 1:**
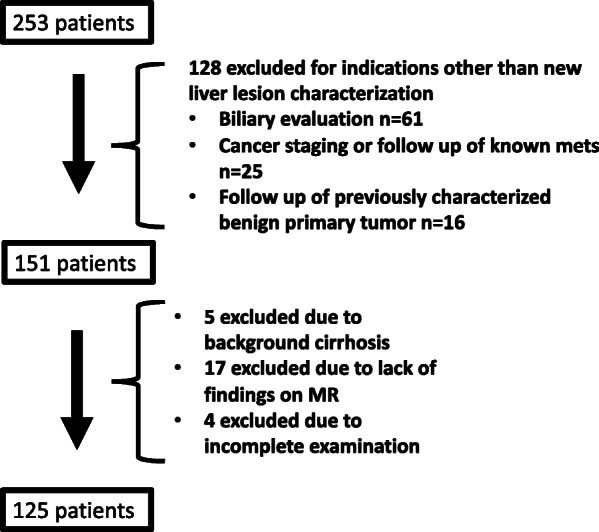
Patient selection flow diagram

We queried our MRI database and identified 253 consecutive patients who underwent MRI with gadoxetic acid over a 5-year period (2008–2013). Of the total patients identified, 128 patients were excluded for indications other than new liver lesion characterization (biliary evaluation [*n* = 61], cancer staging or follow-up of known metastatic disease [*n* = 25] or follow-up of previously characterized benign primary liver tumor [*n* = 16]). Five patients were excluded due to background of hepatic cirrhosis. Seventeen patients were excluded due to lack of findings on gadoxetic acid-enhanced MR imaging and 4 additional cases were excluded due to incomplete MR examination. Therefore, a total of 125 remaining patients were included in the study (Fig. 1). Patient demographics are summarized in Table 1.

**Table 1:** Patient demographics

Demographics	
Age in years (median [range])	46 (20–85)
Female (%)	100 (80%)
Average lesion size	2.6 ± 1.8 cm
Remote history of malignancy (%)	24 (19.2%)

Fourteen patients (11.2%) underwent targeted liver biopsy or surgical resection of the focal liver lesion. Sixty-eight patients (54.4%) had imaging follow-up at least 6 months after the initial MRI (median 43 months [range 6–96]) and 3 patients had imaging follow-up less than 6 months (median 4 months [range 3–5]). Threshold growth was defined as size increase by minimum 5 mm and more than 50% increase in size in less than 6 months or more than 100% increase in size in 6 months or more [13].

### MRI techniques

MR imaging was performed on 1.5T and 3T scanners (GE Healthcare [Chicago, IL] or Siemens [Erlangen, Germany]). Imaging parameters are summarized in Table 2. Unenhanced sequences performed included coronal and axial T2-weighted turbo-spin-echo sequences, axial T1-weighted in- and opposed-phase gradient-echo (GRE) sequence, and axial diffusion-weighted imaging (DWI) with *b* = 50 and *b* =600. Dynamic imaging was performed in the axial plane using 3D T1-weighted fat-saturated GRE sequence.

**Table 2:** Imaging parameters

Sequences	Repetition time (ms)	Echo time (ms)	Flip angle (degrees)	Slice thickness (mm)	Matrix Size	Field of view (cm)
Axial and coronal T2-weighted fast spin echo	1000–1200	75–85	90	4–5	256 × 192 (1.5 T) 288 × 192 (3.0 T)	38–42
Axial T1 dual-echo in and opposed phases, spoiled gradient recall echo (GRE)	170–180	2.2–2.8/4.4–5.3 (1.5 T) 1.1–1.3/2.2–2.6 (3.0 T)	80	6–7	256 × 128–192 (1.5 T) 320 × 160 (3.0 T)	36–40
Axial diffusion-weighted *b* = 50, 600	4400–11250	50–60 (1.5 T) 80–95 (3.0 T)	90	5	320 × 320 (1.5 T) 256 × 256 (3.0 T)	36–40
Axial 3D GRE with fat saturation, post-contrast, including hepatobiliary phase at 20 min	3–4 (1.5T) 4–5 (3.0T)	1–2 (1.5 T) 2–3 (3.0 T)	11–15	3.3–3.6	256 × 176 (1.5 T) 256 × 160 (3.0 T)	36–40

Contrast-enhanced sequences were acquired in the late arterial, portal venous, and interstitial, and delayed phases followed by 20-min delayed HPB phase. A timing run was used to obtain the appropriate delay for the late arterial phase. Portal venous, interstitial, and delayed phases were obtained 45, 9, and 135 s after the arterial phase, respectively. Intravenous contrast was administered either as gadoxetic acid (Eovist, Bayer Pharmaceuticals, Whippany, NJ; [73 patients]) or gadoxetic acid mixed with gadopentetate dimeglumine (Magnevist, Bayer Pharmaceuticals, Whippany, NJ; 52 patients]). Gadoxetic acid was administered at a dose of 0.025 mol/kg body weight when used alone or 0.025 mmol/kg mixed with 0.1 mmol/kg of gadopentetate dimeglumine.

### MRI analysis

Initially, focal liver lesions were identified by an abdominal radiology fellow after reviewing all relevant preceding imaging. The lesions were then labeled by placement of an arrow overlay pointing to the lesion on the imaging sequence where the lesion was best seen, excluding the 20-min delayed HPB phase. If more than one lesion with the same imaging characteristics were present, the largest lesion was chosen for analysis. The labeled image was then saved to a folder on our institutional picture archiving and communication system (PACS; McKesson, Irving, Texas) where the readers could view the indexed liver lesion and access the other sequences from that particular MRI examination.

Two board-certified fellowship-trained abdominal radiologists, each with 4 years of faculty experience in reading body MRI studies, then retrospectively evaluated the MRI studies of all patients in two separate sessions at least 4 weeks apart. In the first session, the readers were asked to evaluate liver lesions using only conventional unenhanced sequences and contrast-enhanced dynamic phase images (late arterial, portal venous, interstitial, and delayed phase). In the second session, the readers were asked to evaluate liver lesions using all sequences including the 20-min delayed HPB phase images. Both readers were blinded to the clinical information except for history of malignancy. Characterization of liver lesions was based on personal experience of the respective reader. Response forms were constructed using Google Forms survey software (Google, Mountain View, CA). The readers were asked to provide a diagnosis and rate their confidence in the diagnosis using a scale ranging from 0 to 3; a value of 0 signified complete uncertainty, 1 low certainty, 2 moderate certainty, and 3 high certainty. All responses were collected electronically with documentation of date and time of submission.

### Statistical analysis

Inter-reader agreement in diagnosis was assessed using Cohen κ statistics. Change in diagnosis and confidence in diagnosis before and after assessment of the 20-min delayed HPB phase images were calculated using the Wilcoxon signed rank test. In the 14 patients who underwent subsequent targeted liver biopsy or surgical resection, histopathology was used as reference standard to calculate change in diagnostic accuracy using the Mcnemar test. All statistical analyses were performed using Stata software v.14.1 (StataCorp, College Station, TX).

## Results

There was no significant change in diagnosis before and after evaluating the HPB phase for both readers (*p* = 1.0 for reader 1 (R1); *p* = 0.34 for reader 2 (R2)). Confidence in diagnosis decreased from average 2.8 ± 0.45 to 2.6 ± 0.59 for R1 and increased from 2.6 ± 0.83 to 2.8 ± 0.46 for R2. Change in confidence was statistically significant for R1 (*p* = 0.003) but not significant for R2 (*p* = 0.49). Inter-reader agreement in diagnosis was good without (*k* = 0.66) and with (*k* = 0.75) incorporation of the HPB phase images.

Reader characterization of lesions before and after evaluation of the HPB phase and change in confidence are summarized in Tables 3 and 4, respectively. There was no significant change in diagnosis of each individual lesion category after evaluating the HPB phase. In particular, there was no significant change in diagnosis of FNH or adenoma for both readers (FNH: R1, *p* = 0.58 and R2: *p* = 0.54; adenoma: R1, *p* = 0.06 and R2, *p* = 1.0). Case-matched comparison of confidence in diagnosis was also not statistically different for each lesion category including both FNH and adenoma (FNH: R1, *p* = 1.0 and R2, *p* = 0.12; adenoma: R1, *p* = 1.0 and R2, *p* = 0.62).

**Table 3:** Reader characterization of lesions before and after evaluation of the HPB phase and change in confidence

	Reader 1 Before HPB phase	Reader 1 After HPB phase	*p* value	Reader 2 Before HPB phase	Reader 2 After HPB phase	*p* value
FNH	59	57	0.58	49	53	0.54
Adenoma	10	17	0.06	17	16	1.0
Hemangioma	24	20	0.12	22	25	0.54
Metastasis	16	18	0.50	15	13	0.62
Biliary hamartoma	2	3	1.0	2	2	1.0
Perfusional abnormality	6	3	0.45	7	4	0.25
Abscess	1	1	1.0	1	1	1.0
Focal fat	3	3	1.0	2	2	1.0
Angiomyolipoma	1	1	1.0	1	1	1.0
Dysplastic nodule	1	1	1.0	0	0	NA
HCC	1	0	1.0	0	0	NA
Indeterminate	1	1	1.0	8	8	1.0
Lymphoma	0	0	NA	1	0	1.0

**Table 4:** Reader characterization of lesions before and after evaluation of the HPB phase and change in confidence

	Reader 1 Avg. confidence Before HPB phase	Reader 1 Avg. confidence After HPB phase	*p* value	Reader 2 Avg. confidence Before HPB phase	Reader 2 Avg. confidence After HPB phase	*p* value
FNH	2.8	2.6	0.2	2.9	2.8	0.4
Adenoma	2.8	2.4	0.1	2.9	2.9	1.0
Hemangioma	2.8	2.7	1.0	2.4	2.8	0.5
Metastasis	3.0	2.9	0.5	2.9	2.9	1.0
Biliary hamartoma	3.0	3.0	1.0	3.0	3.0	1.0
Perfusional abnormality	3.0	2.3	0.2	2.4	2.4	1.0
Abscess	2.0	2.0	1.0	3.0	3.0	1.0
Focal fat	2.7	3	1.0	3.0	3.0	1.0
Angiomyolipoma	3.0	3.0	1.0	3.0	3.0	NA
Dysplastic nodule	2.0	0	NA	–	–	NA
HCC	1.0	1.0	1.0	–	–	NA
Lymphoma	–	–	NA	3.0	–	NA

Sub-group analysis based on contrast agent mixture did not demonstrate a significant change in diagnosis when gadoxetic acid was used alone (73 patients, *p* = 1.0 for R1; *p* = 1.0 for R2) versus gadoxetic acid mixed with gadopentetate dimeglumine (52 patients, *p* = 1.0 for R1; *p* = 0.13 for R2). There was a decrease in confidence for R1 in cases where gadoxetic acid was mixed with gadopentetate dimeglumine (*p* = 0.03) while confidence was not significantly changed when gadoxetic acid was used alone (*p* = 0.09). There was no change in confidence for R2 based on contrast agent mixture (*p* = 0.48 with gadoxetic acid alone; *p* = 1.0 with gadoxetic acid was mixed with gadopentetate dimeglumine).

**Fig 2.**
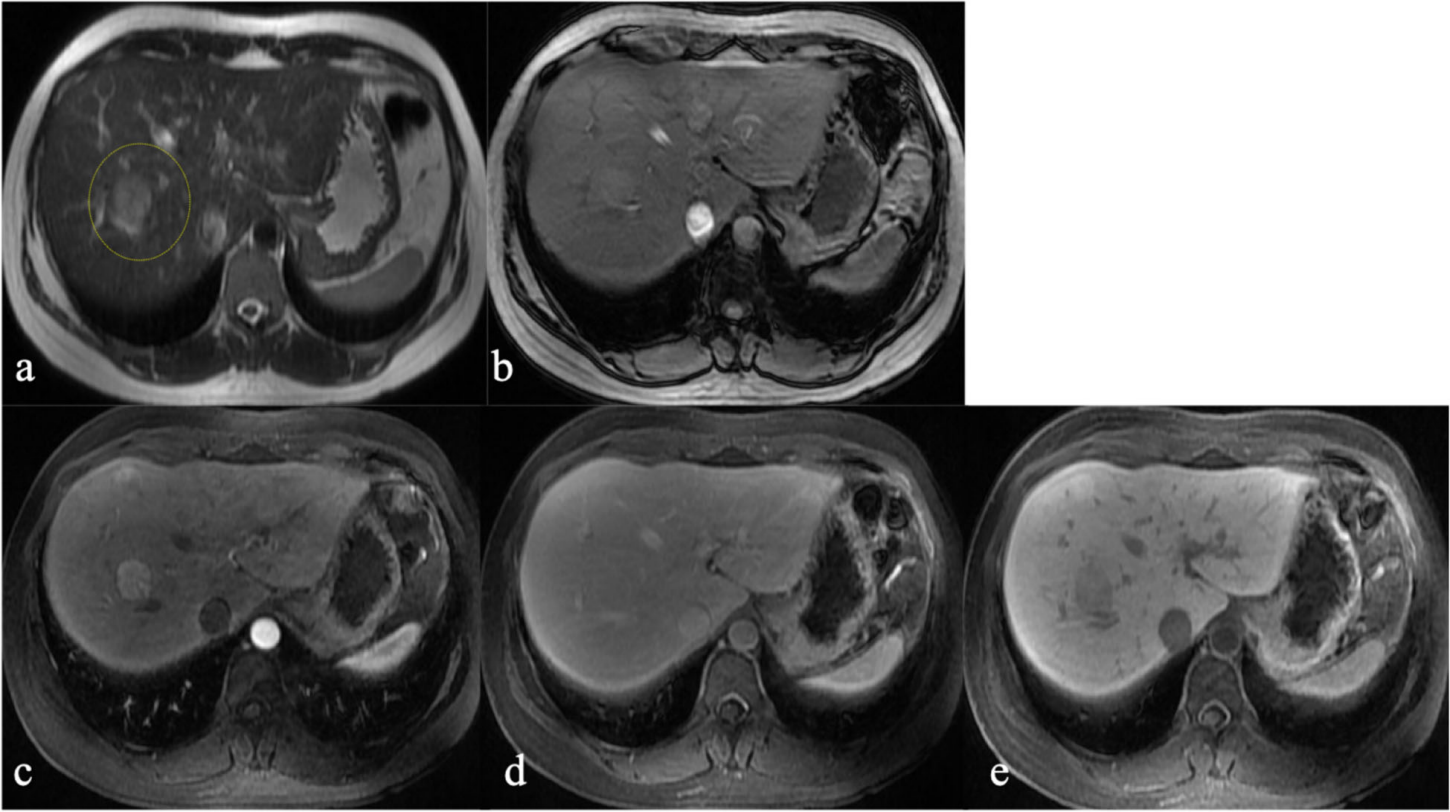
31-year-old female with pathologically proven FNH. Axial T2W (**a**) and axial T1W opposed-phase (**b**) prior to surgery demonstrate a heterogeneous mildly T2 hyperintense lesion in segment III (yellow dotted circles in **a**) with no internal fat. Gd-EOB-DTPA-enhanced axial T1W fat-saturated sequences demonstrate heterogeneous arterial enhancement (**c**), iso-intense signal in the portal-venous phase (**d**), and retention of contrast in the HPB phase (**e**). R1 initially classified this lesion as adenoma but correctly reclassified as FNH after assessment of the HPB phase. R2 correctly classified this lesion as FNH before and after assessment of the HPB phase. Confidence in diagnosis increased from moderate to high certainty for R2 and remained at high certainty for R1

**Fig 3:**
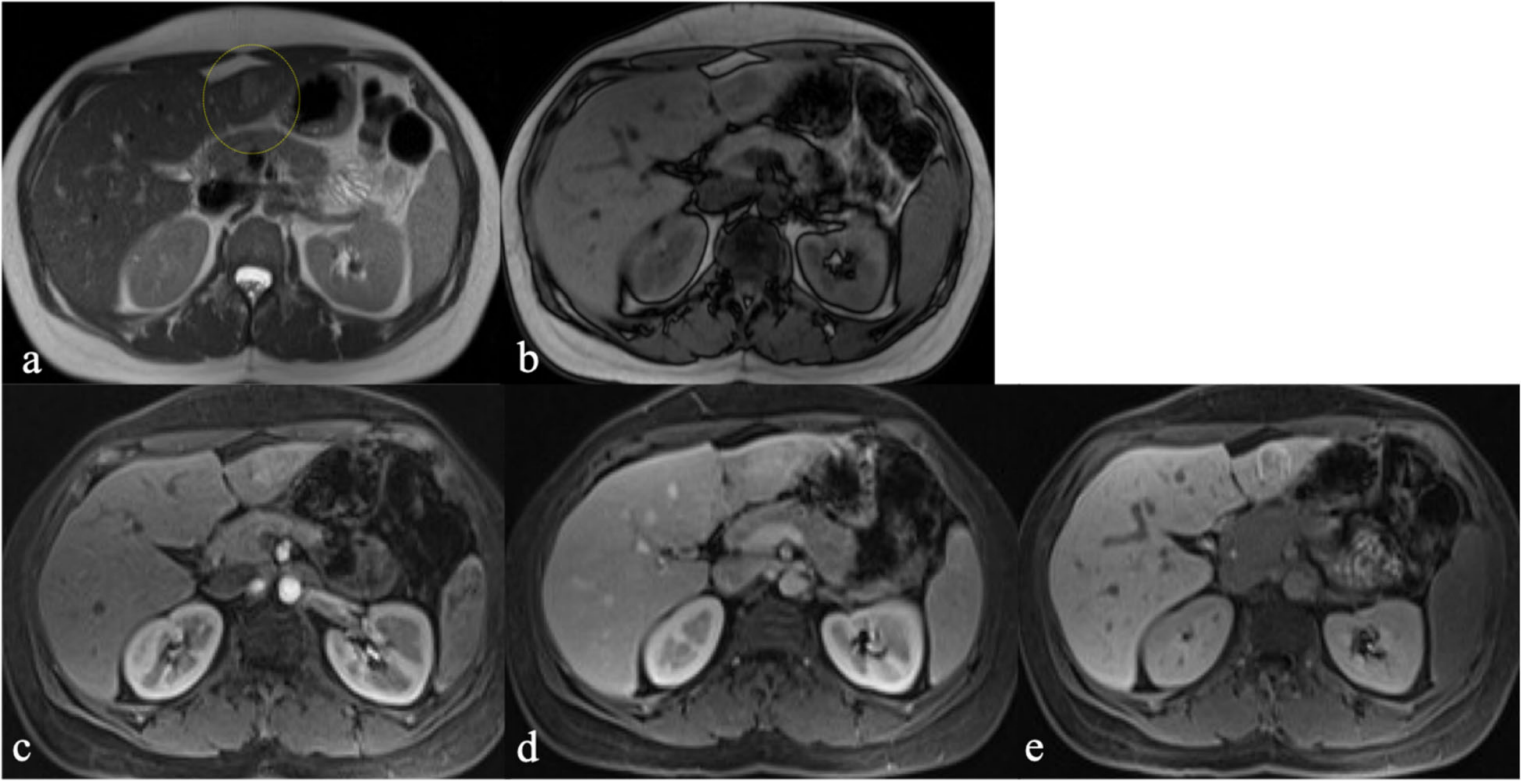
30-year-old female with pathologically proven hepatic adenoma. Axial T2W (**a**) and axial T1W opposed-phase (**b**) prior to surgery demonstrate a T2 hyperintense lesion in segment VIII (yellow dotted circles in **a**) with no internal fat. Gd-EOB-DTPA-enhanced axial T1W fat-saturated sequences demonstrate arterial enhancement (**c**), iso-intense signal in the interstitial phase (**d**), and mild heterogeneous retention of contrast in the HPB phase (**e**). Both readers classified this lesion as FNH before and after assessment of the HPB phase. Confidence in diagnosis increased from moderate to high certainty for R1 and remained at high certainty for R2

The 14 lesions for which histopathology was available were determined to be metastasis (*n* = 8), adenoma (*n* = 3), FNH (*n* = 2), or biliary hamartoma (*n* = 1). R1 correctly characterized 10/14 lesions before and 11/14 lesions after evaluating the HPB phase (*p* = 1.0). R2 correctly characterized 10/14 lesions before and 9/14 lesions after evaluating the HPB phase (*p* = 0.16). With the addition of the HPB phase, R1 correctly changed one diagnosis from adenoma to FNH (Fig. 2); R2 correctly changed one diagnosis from hemangioma to metastasis but incorrectly changed one diagnosis from FNH to hemangioma and one metastasis to “indeterminate.” Both readers incorrectly diagnosed 3 lesions before and after assessing the HPB phase including 2 adenomas which were characterized as FNH (Fig. 3) and 1 biliary hamartoma which was characterized as metastasis.

On follow-up imaging in 68 of 125 patients, 48 lesions remained stable and 16 decreased in size. Out of four patients in whom lesions increased in size on follow-up imaging, two had remote history of malignancy, and both of these lesions were characterized as metastasis by both readers. The other two lesions were characterized as FNH and hemangioma by both readers.

## Discussion

In our study, we demonstrated that addition of the 20-min delayed HPB phase in gadoxetic acid-enhanced MR imaging did not significantly change the diagnosis for both expert readers when characterizing newly discovered focal liver lesions in non-cirrhotic patients. Average confidence in diagnosis decreased for reader 1 and increased for reader 2 but the overall change in confidence category was only statistically significant for reader 1. In lesions for which histopathology was available, there was no statistical difference in the number of correctly characterized lesions without and with evaluation of the HPB phase.

Our study focused on whether the HPB phase adds any value in characterizing newly discovered liver lesions in non-cirrhotic patients. We included a range of unknown lesions referred for gadoxetic acid-enhanced MR imaging reflecting what is seen in clinical practice. The results of our study demonstrated that there was no clear benefit of obtaining gadoxetic acid-enhanced MR in evaluating these lesions. HPB phase of gadoxetic acid-enhanced MR imaging has been shown to improve detection and characterization of lesions which do not have functioning hepatocytes and bile ducts [14, 15]. In our study, the information obtained from the HPB phase did not significantly change readers’ decision on diagnosis compared with information from conventional and dynamic post-contrast sequences. A previous study by Haimerl et al. reported that HPB phase of gadoxetic acid-enhanced MR improved differentiation of focal solid hepatic lesions [16]. However, this study included both malignant and benign lesions including only 20 benign primary liver lesions. Our results are in agreement with previous reports evaluating gadoxetic acid-enhanced MR imaging in characterization of primary liver tumors in non-cirrhotic patients [11, 12]. In their series of 29 patients, Donati et al. reported correct diagnosis 66–76% before and 66–79% after evaluation of the HPB phase comparable to 71% before and 64–79% after the HPB phase in our study. Similarly, Purysko et al. reported no statistically significant difference in making the correct diagnosis before (79-94%) and after (96–100%) evaluation of the HPB phase in their series of 47 patients. To our knowledge, our study is the largest of its kind and also the first to include patients who had received gadoxetic acid mixed with gadopentetate dimeglumine and showed no significant change in diagnosis based on contrast mixture.

**Fig 4.**
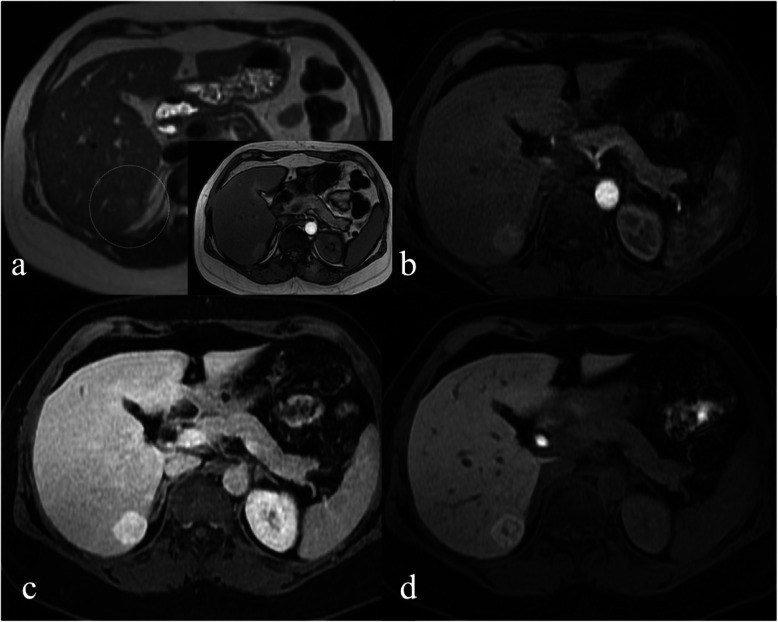
46-year-old female with an incidentally detected liver lesion. Axial T2W (**a**) demonstrates a heterogeneous mildly T2 hyperintense lesion in segment VII (dotted circle in **a**) with no internal fat on axial T1W opposed-phase (inset). Subtracted Gd-EOB-DTPA-enhanced axial T1W fat-saturated sequences demonstrate arterial enhancement (**b**), persistent high signal in the delayed phase (**c**) and retention of contrast in the HPB phase (**d**). R1 characterized this lesion as FNH before and after assessment of HPB phase. R2 initially classified this lesion as adenoma but reclassified as FNH after assessment of the HPB phase. Confidence in diagnosis decreased from high to moderate certainty for R2 and remained at high certainty for R1

A common indication for gadoxetic acid-enhanced MR imaging of incidentally detected liver lesion is differentiating FNH and HCA [3, 8]. Gadoxetic acid is taken up by functioning hepatocytes and excreted in biliary ductules. FNH is expected to accumulate HPB-specific contrast agents since it is composed of functioning hepatocytes with abnormal biliary ductules [17] whereas HCA would not as it has been thought of as lacking functioning hepatocytes and bile ducts [2]. However, the inflammatory subtype of HCA, which accounts for up to half of cases of HCA, can mimic FNH on both conventional and gadoxetic acid-enhanced MR imaging [3, 18, 19]. Both FNH and inflammatory HCA typically demonstrate mild hyperintense signal intensity on T2-weighted imaging and arterial phase hyperenhancement that persists in the portal venous and delayed phases [20]. Intra-lesional fat is seen in only a minority of inflammatory HCA subtypes (range, 10–20%) [21, 22]. Similar to FNH, inflammatory HCA can show gadoxetic acid retention and therefore appear iso- or hyperintense in the HPB phase [18]. Retention of gadoxetic acid in the HPB phase in both FNH and HCA has been attributed to the presence of sinusoidal dilatation and/or expression of the organic anionic transport protein (OATP1) [18, 23]. Similar rate of iso- or hyperintensity has also been reported with the use of gadobenate dimeglumine-enhanced MR imaging [24]. One proposed distinguishing MR feature of HCA is the presence of peripheral high signal intensity on T2-weighted imaging (“atoll” sign) which has been reported in 50–80% of cases [20, 22]. In addition, a recent systematic review found that there has been inconsistent reporting of histopathologic HCA subtypes in previously published diagnostic accuracy studies which may have resulted in incorrect pathologic classification of some inflammatory HCA subtypes as FNH leading to overestimation of diagnostic accuracy of gadoxetic acid-enhanced MR imaging in differentiating FNH from HCA [9]. In our study, diagnosis of FNH and HCA and confidence in diagnosis was not significantly different before and after evaluation of the HPB phase. There were seven more diagnoses of HCA for R1 after evaluation of the HPB phase although this did not reach statistical significance. These findings perhaps reflect the readers’ awareness of the pitfalls of gadoxetic acid-enhanced MR in distinguishing FNH and HCA (Fig. 4).

While the absolute change in average confidence in diagnosis was similar for both readers (0.2 decrease for R1 and 0.2 increase for R2), there was a statistically significant decrease for R1 since R1’s confidence in diagnosis was more frequently changed from high certainty (confidence level 3) to low certainty (confidence level 1) after evaluation of the HPB phase while R2’s confidence more frequently changed from indeterminate (confidence level 0) to either low or high certainty (confidence level 1 and 3, respectively). In their series of 29 patients, Donati et al. reported that reader confidence increased in 2 of 3 readers after evaluation of HPB phase without any significant change in correctly diagnosed lesions [11]. Similarly, Purysko et al. demonstrated that despite no change in diagnosis, reader confidence increased in 1 of 3 readers in diagnosing HCA and in 2 of 3 readers in diagnosing FNH [12]. The confidence levels in our study could not be directly compared with that previously reported by Donati et al. and Purysko et al. since different confidence level scales were used in each study. The increase in reader confidence in prior reports was attributed to the characteristic enhancement pattern of FNH in the HPB phase [11, 12]. The variable reader confidence levels in our study may be partly explained by a wider range of lesions included in our study and partly by an inherent reader bias when evaluating the HPB phase. Interestingly, sub-group analysis based on contrast mixture demonstrated that confidence level for R1 decreased in cases where gadoxetic acid was mixed with gadopentetate dimeglumine compared to cases where gadoxetic acid was used alone which was reflected in the overall confidence level of R1 after evaluation of the HPB phase. This may be explained in part by hepatobiliary excretion of gadoxetic acid by healthy hepatic parenchyma even during the early postcontrast dynamic series, resulting in a relative loss of signal intensity contrast between the hepatic parenchyma and enhancing lesions.

Our study has several limitations. First, this was a retrospective study. Second, histopathology was not available in the majority of cases. This is expected since lesions which are determined to be benign on MRI rarely undergo biopsy. Follow-up imaging was performed in over half of the patients in this study (52.1% [64/125]), and the vast majority of those lesions remained stable 6 months or later (93.7% [60/64]). Of the four lesions which increased in size, the first was characterized as FNH and the second as hemangioma by both readers with high certainty before and after evaluating the HPB phase. The remaining two lesions were categorized as metastases by both readers with high certainty before and after evaluating the HPB phase. Finally, change in diagnosis was not weighed based on level of confidence.

## Conclusions

In conclusion, our results suggest that the routine use of gadoxetic acid-enhanced MR for characterization of newly discovered indeterminate lesions in non-cirrhotic patients may not be necessary as the information obtained from the HPB phase does not necessarily change the diagnosis or confidence in diagnosis.

## Data Availability

The datasets used and/or analyzed during the current study are available from the corresponding author on reasonable request.

## Abbreviations

CT: Computed tomography
DWI: Diffusion-weighted imaging
FNH: Focal nodular hyperplasia
GRE: Gradient Echo
HCA: Hepatocellular adenoma
HIPPA: Health Insurance Portability and Accountability Act
HPB: Hepatobiliary phase
MRI: Magnetic resonance imaging
PACS: Picture archiving and communication system
US: Ultrasound
